# Intentional creation of suboptimal, realistic dose distributions

**DOI:** 10.1002/acm2.70305

**Published:** 2025-10-27

**Authors:** Skylar S. Gay, Mary P. Gronberg, Raymond Mumme, Beth M. Beadle, Anuja Jhingran, Tze Yee Lim, Zhiqian H. Yu, Christine Chung, Meena Khan, Chelsea Pinnix, Sanjay Shete, Brent Parker, Tucker J. Netherton, Carlos E. Cardenas, Laurence E. Court

**Affiliations:** ^1^ Department of Radiation Physics The University of Texas MD Anderson Cancer Center Houston Texas USA; ^2^ The University of Texas MD Anderson Cancer Center UTHealth Houston Graduate School of Biomedical Sciences Houston Texas USA; ^3^ Department of Radiation Oncology UT Southwestern Medical Center Dallas Texas USA; ^4^ Stanford University Stanford California USA; ^5^ Department of Radiation Oncology The University of Texas MD Anderson Cancer Center Houston Texas USA; ^6^ Department of Biostatistics The University of Texas MD Anderson Cancer Center Houston Texas USA; ^7^ Department of Radiation Oncology The University of Texas Medical Branch Galveston Texas USA; ^8^ Department of Radiation Oncology The University of Alabama at Birmingham Birmingham Alabama USA

**Keywords:** education, plan quality

## Abstract

**Background:**

Radiation oncology residents report a lack of understanding and confidence in assessing radiotherapy plan quality. A contributing factor is the environment in which plan review is taught during residency, that is, routine clinical practice, which does not provide ample time for self‐guided practice in a low‐stakes setting. Expertise in plan review requires diverse case presentation and many examples, which are often not achievable in smaller programs and for less common cancer types. As plan quality affects patient outcomes, it is important to address these pitfalls in the education of residents on plan review.

**Purpose:**

To address the identified pitfalls of clinic‐based training, we have developed techniques to create realistic dose distributions that appear suboptimal in a controllable way. These plans can provide many more case examples in the training curriculum and present a low‐stakes technique for safe and effective education of radiation oncology residents.

**Methods:**

High‐quality dose distributions were first generated with a pre‐trained deep learning model (trained using only high‐quality plans). The dose distributions were then altered directly to create three classes of suboptimal dose distributions: (1) decreased organ‐at‐risk sparing, (2) decreased target conformality, and (3) hotspots in the target. Experienced clinicians then reviewed a subset of these suboptimal dose distributions to assess realism.

**Results:**

We successfully decreased the quality of radiotherapy dose distributions. The decreased organ‐at‐risk sparing, decreased target conformality, and increased target hotspots were statistically significant (*p* < 0.05) when assessed by dose‐volume histogram metrics for all parameters evaluated, and the magnitude of dose change was controllable. The resulting dose distributions were overall scored by experienced clinicians as realistic.

**Conclusion:**

In this study, we developed techniques to generate realistic but suboptimal dose distributions. The techniques operate directly on existing dose distributions without the need for a treatment planning system and produce dose distributions that appear realistic to experienced clinicians.

## 1 INTRODUCTION

Despite extensive training during a radiation oncology residency, residents report substantial lack of confidence in their plan review skills and understanding of plan quality.^1^ Several pitfalls in the current clinic‐based training contribute to this issue. Many clinics encounter limited examples of disease sites; for example, 25% of academic centers and 55% of community centers in the United States treat fewer than four head and neck (HN) cancer cases annually (unpublished National Cancer Database analysis, 2021). Even for more common disease sites, residents are typically exposed to only a single treatment plan instead of the many different plans that are possible and may need improvement. Finally, the clinic itself is a high‐stakes environment. Current patient plans are reviewed with the residents as part of their training, and there is substantial time pressure to approve plans for treatment rather than allocate time for education.

These pitfalls limit effective learning and can propagate to deficiencies in future clinical practice. In a typical clinical workflow, radiation oncologists provide the penultimate reviews of plan quality and approval. Unfortunately, the literature shows that lower‐quality plans make their way to patient delivery,^2^, ^3^ with a recent study reporting that 45% of suboptimal plans were approved for treatment delivery even after undergoing the peer review process.^4^ As it is known that plan quality affects patient outcomes, addressing shortcomings in the educational processes for plan review could lead to improved patient care.

One approach to improve resident plan review education might be to curate a database of low‐quality plans from multiple institutions.^5^, ^6^ However, the use of curated databases for long‐term education has limitations, such as the possibility of inadvertent memorization of suboptimal plans by trainees or advances in treatment techniques that render the training examples obsolete. It would instead be ideal to generate training examples directly. While there has been substantial effort in automatically generating high‐quality dose distributions for therapy,^7^, ^8^, ^9^, ^10^, ^11^ to date, little attention has been given to intentionally generating suboptimal dose distributions for education.

Therefore, this study introduces methods to generate suboptimal dose distributions that are realistic. The magnitude and geometric extent of suboptimal features are fully controllable, enabling generation of both grossly suboptimal training examples and more subtle errors requiring only minor corrections, thus providing opportunities for a thorough education. These new suboptimal dose distributions can be introduced into the educational curriculum, allowing for comprehensive plan review practice through many training examples.

## 2 METHODS

In this study, suboptimal plans were modeled as changes to the dose distributions of predicted volume‐modulated arc therapy (VMAT) plans. These dose distributions were first predicted with the three‐dimensional densely dilated U‐Net model^7^ using model ensembles for HN^12^ and gynecological (GYN)^13^ treatment sites. Each ensemble had been previously trained using three‐fold cross‐validation, where the training data had been carefully curated to be high‐quality using feedback from experienced clinicians and dose‐volume histogram metrics; and each have been demonstrated to produce high‐quality dose distributions. Separate techniques were then used to modify these predicted dose distributions to generate three different types of suboptimal dose distributions: (1) increasing the dose to organs at risk (OARs) to simulate poor OAR sparing, (2) decreasing the dose around targets to simulate poor target conformality, and (3) adding high‐dose regions into the target to simulate hotspots within the target. Each technique is described in greater detail below.

### 2.1 Treatment sites and data sets

Two treatment sites were investigated in this study. A set of 39 HN VMAT cases was curated from a comprehensive cancer center. These cases included a planning computed tomography (CT) data set, planning target volume (PTV) contours prescribed to varying dose levels, and OAR contours required to generate high‐quality dose predictions.^12^ A second data set of 20 GYN VMAT cases was curated from the same comprehensive cancer center. These cases included a planning CT data set, a single PTV prescribed to 4500 cGy, and OAR contours required to generate high‐quality dose predictions.^13^

### 2.2 Geometrically aware convolution

When changing voxels in a dose distribution, neighboring dose values must be taken into account. Furthermore, it is important that the geometric extent of the changes is controllable; for example, an increase in OAR dose to simulate poor sparing should affect only OARs which are believably in or near the beam delivery path. To address this issue, we developed a geometrically aware convolution method and used it for the first two suboptimal dose generation techniques. This allows for updates that incorporate neighboring dose voxel values and restrict changes to a localized region.

The geometrically aware convolution modifies the dose distribution with a kernel that constantly updates its parameters based on its position within the patient anatomy and applies a scaling parameter to the existing dose values (Equation 1).

Equation 1: Function to provide a geometrically‐aware kernel. g(λ,a): function describing the geometrically aware kernel. s(λ,a): linear scaling function applied to control overall kernel scaling. k(λ): receptive field. λ: three‐dimensional geometric feature. a: maximum dose scaling parameter.

(1) $$g\left(\lambda ,a\right)=s\left(\lambda ,a\right)\cdot \frac{1}{\sum k}k\left(\lambda \right)$$

The position within the patient anatomy is described by a geometric feature array, λ, that incorporates both Euclidean distances from structures and angular distances from a ray originating outside the body and terminating in the target. The resulting three‐dimensional array has identical dimensions to the predicted dose array. Each voxel of λ is used by the kernel to set its parameters when modifying the corresponding dose voxel. The specific generation steps of λ and the dimensions of the receptive field k(λ) are different for Techniques 1 and 2 and are described in greater detail in the relevant sections below.

The kernel scaling to the dose distribution also varies based on geometry so that the greatest scaling will only be applied to the desired regions. This is provided by a linear function (Equation 2) and relies on geometric feature λ as well as a scaling parameter, a, that controls the upper bound of the scaling when increasing the dose (Technique 1) or the lower bound when decreasing the dose (Technique 2). To ensure that only the linear function changes the scale of the dose values, the parameters of the receptive field k(λ) are normalized to sum to 1.

Equation 2: Function to vary dose scaling with respect to geometry. s(λ,a): geometrically aware linear scaling function. λ: three‐dimensional geometric feature. a: the maximum dose scaling parameter.

(2) $$s\;\left(\lambda ,a\right)=\left(1-a\right)\;\lambda +a$$

The convolution of the high‐quality dose distribution with this geometrically aware kernel produces a new dose distribution. To ensure visual realism, two post‐processing steps are applied. First, and most notably for small structures when a small scaling value is used (e.g., a=1.1), the dose is occasionally modified opposite to the desired effect when nearby dose values are smoothed into the structures by the kernel. Therefore, a voxel‐wise maximum (for Technique 1) or minimum (for Technique 2) function that compares the new suboptimal dose distribution to the original ensures that dose values never change inappropriately (Equation 3).

Equation 3: Prevention of dose updates opposite to the desired effect. $D^{\prime }$: the suboptimal dose distribution. D: the dose distributions of predicted volume‐modulated arc therapy f: an element‐wise maximum (Technique 1: Increase OAR Dose) or minimum (Technique 2: Reduce Target Conformality) function. g(λ,a): function describing the geometrically aware kernel. λ: three‐dimensional geometric feature. a: the maximum dose scaling parameter.

(3) $$D^{\prime }=f\left(D*g\left(\lambda ,a\right),D\right)$$

Second, unrealistic hotspots outside the target boundary were sometimes introduced when increasing the dose to OARs, particularly for structures near the target such as parotids (HN cases) or femoral heads (GYN cases). Therefore, a final upper threshold is applied so that all dose values greater than 104% of the prescription dose are reset to 98% of their value. This is only applied to dose distributions generated by Technique 1.

Equation 4: Technique to prevent out‐of‐target hotspots in the suboptimal dose distributions. D’: the suboptimal dose distribution output from Equation 3. d: individual voxel dose values in D’. D_final_: the final suboptimal dose distribution. Rx: the prescription dose level.

(4) $$D_{final}=\left\{\begin{array}{c} d\;if\;d\leq 1.04\;Rx\\ 0.98\times d \end{array}\right.\;\;\forall \;d\in D^{\prime }$$

### 2.3 Technique 1: Increase OAR dose

To increase the dose to an OAR, a scaling factor a>1 is selected. The geometric feature ${\lambda_{OAR}}_{n}$ for a given OAR consists of two components: (1) the Euclidean distances from the OAR boundary to all voxels outside (denoted $\rho_{OAR_{n}}$) and (2) voxel‐wise angular distances from a ray passing through the centers‐of‐mass of the OAR and terminating on the target centers‐of‐mass (denoted $\phi_{OAR_{n}}$). The centers‐of‐mass from both the OAR and the target are found on a slice‐by‐slice basis to account for the varying shapes in the z‐dimension. In addition, for HN cases that have multiple targets, a union structure of the targets is used. To prevent excessive angle‐based modifications to the dose beyond the extent of the target and OAR, the angular feature is also restricted to only include axial slices containing the OAR and/or the target, with a three‐slice margin above and below.

Both components are normalized to the range [0, 1], using only values within the patient anatomy and with all values outside reset to 1. To restrict dose changes to be reasonably near the OAR, they are then fit to a sigmoid‐like distribution (Equation 5). The parameters fwhm=0.05 and β=5 were experimentally determined to produce realistic results.

Equation 5: Function to generate a sigmoid‐like distribution. λ: a three‐dimensional geometric feature ranging from 0–1. fwhm: the value for the full‐width half‐max of the distribution. β: a parameter controlling the steepness of the distribution.

(5) $$f\;\left(\lambda ,fwhm,\beta \right)=\frac{{\left(\lambda^{-\frac{\log2}{\log fwhm\;}}\right)}^{\beta }}{{\left(\lambda^{-\frac{\log2}{\log fwhm}}\right)}^{\beta }+{\left(1-\;\lambda^{-\frac{\log2}{\log fwhm\;}}\right)}^{\beta }}$$

The geometric feature for a given OAR, $\lambda_{OAR_{n}}$ (Equation 6), is then simply the weighted voxel‐wise sum of the distance and angular components fit to a sigmoid‐like distribution (Equation 5), where the weighting value of 0.3 applied to the angular component was experimentally determined to produce realistic results. If the dose is to be changed to more than one OAR, individual geometric features are combined by taking a voxel‐wise minimum (Equation 7).

Equation 6: Combination of distance and angular components to generate the geometric feature for a single OAR. $\rho_{OAR_{n}}$: the distance component. $\phi_{OAR_{n}}$: the angular component.

(6) $$\lambda_{OAR_{n}}=\rho_{OAR_{n}}+0.3\;\phi_{OAR_{n}}$$

Equation 7: Combination of individual OARs’ geometric features into the overall OAR geometric feature. $\lambda_{OAR_{n}}$: the geometric feature for a single OAR. $\lambda_{OAR}$: the combined overall OAR geometric feature.

(7) $$\lambda_{OAR}=\min\;\left(\lambda_{OAR_{n}}\;\forall n\in \left\{OARs\right\}\right)$$

Separately, it is important that the dose to the targets not be unrealistically increased. A similar PTV geometric feature denoted $\rho_{PTV}$, the voxel‐wise Euclidean distances within the union of targets’ structure (HN cases) or single target's structure (GYN cases) to the structure boundary, is found and fit to a sigmoid‐like distribution (Equation 5) using the parameters fwhm=0.09 and β=5, as these values were experimentally determined to produce realistic results. The PTV geometric feature is normalized to the range [0, 0.98], which allows realistic and relatively small changes to be made to the dose values near and within the target.

**FIGURE 1:**
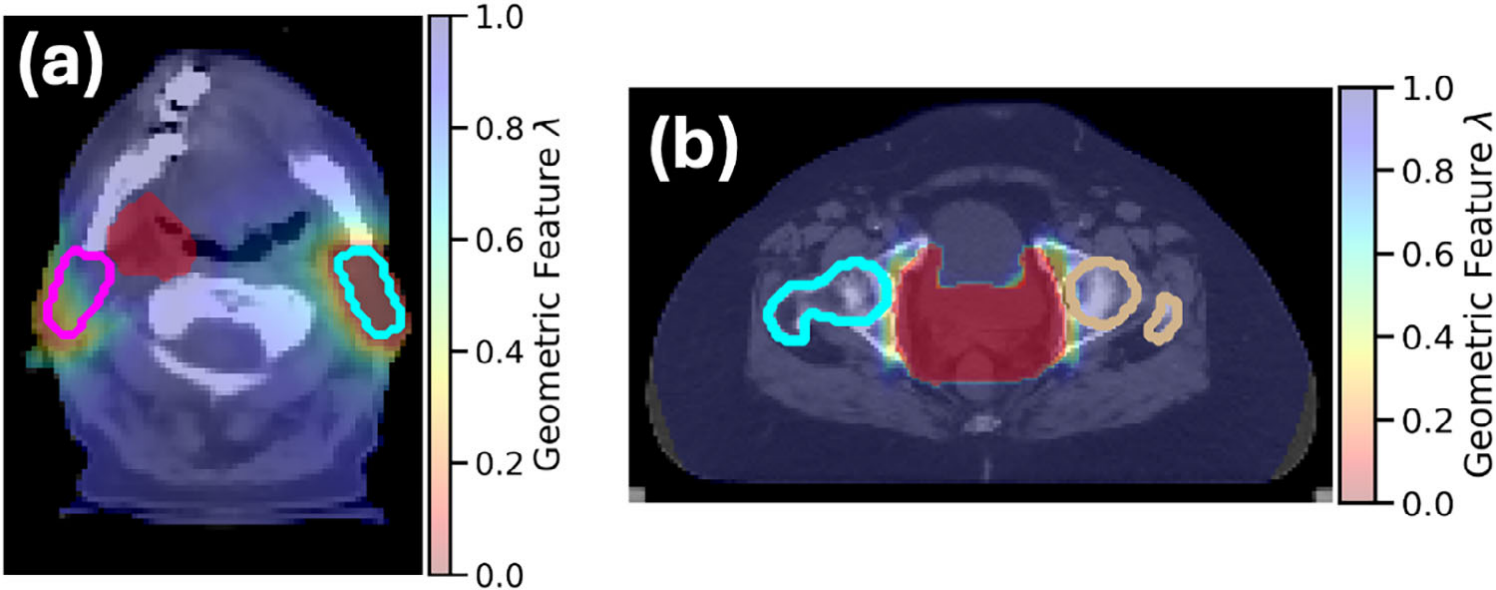
The final geometric feature maps provided for geometrically aware convolution. (a) The feature map for increasing the dose to the parotids in a head and neck case. Cyan and magenta contours: left and right parotids, respectively. Red solid: target prescribed to 7000 cGy. (b) The feature map for reducing target conformality most strongly near the left and right femoral heads in a gynecologic case. The thin ring‐like feature map surrounding the target may also be seen along the anterior and posterior aspects of the target. Tan and cyan contours: left and right femoral heads, respectively. Red solid: target prescribed to 4500 cGy.

The final geometric feature (Equation 8) is the voxel‐wise maximum of the OAR geometric feature $\lambda_{OAR}$, including both distance and angular components (Equation 7), and the PTV geometric feature $\rho_{PTV}$, which only considers distance. An example for an HN case is shown in Figure 1(a).

Equation 8: Generation of the final geometric feature for reducing OAR dose. $\lambda_{OAR}$: the geometric feature for the OARs. $\rho_{PTV}$: the geometric feature for the PTV.

(8) $$\lambda =\max\;\left(\lambda_{OAR},\rho_{PTV}\right)$$

The kernel itself is a (1×5×5) array in the z‐, y‐, and x‐dimensions, respectively. The receptive field k(λ) is described by Equation 9.

Equation 9: Receptive field for the kernel used to increase dose to OARs. λ: geometric feature.

(9) $$k\;\left(\lambda \right)=\left[\begin{array}{ccccc} 1-\lambda & 1-\lambda & 1-\lambda & 1-\lambda & 1-\lambda \\ 1-\lambda & 1-\lambda & 1-\lambda & 1-\lambda & 1-\lambda \\ 1-\lambda & 1-\lambda & 1 & 1-\lambda & 1-\lambda \\ 1-\lambda & 1-\lambda & 1-\lambda & 1-\lambda & 1-\lambda \\ 1-\lambda & 1-\lambda & 1-\lambda & 1-\lambda & 1-\lambda \end{array}\right]$$

For HN cases, the maximum scaling values a=1.1,1.2,1.3,1.4,1.5,1.6,and1.7 were used to generate different dose distributions corresponding to OAR dose increases between approximately 110% and 170%. For GYN cases, the maximum scaling values a=1.1,1.2,1.3,1.4,1.5,and1.6 were used, with a=1.7 excluded as it was observed to result in unrealistically hot dose distributions.

In this study, increases to the OAR dose were investigated in groups of one or more OARs. Groupings were made based on geometric or dosimetric relevance and with clinician guidance. Five OAR groups (esophagus and larynx, brainstem, optic nerves and lenses, parotids, and cochleae) were investigated for HN cases, and three OAR groups (bladder, rectum, and femoral heads) were investigated for GYN cases.

For the initial 39 HN cases, 1274 suboptimal dose distributions with reduced OAR sparing were generated across the seven scaling parameters and five OAR groups. For the initial 20 GYN cases, 348 suboptimal dose distributions with reduced OAR sparing were generated across the six scaling parameters and three OAR groups.

### 2.4 Technique 2: Reduce target conformality

To decrease PTV conformality resulting from overly sparing an OAR, a scaling factor a<1 is selected. The geometric feature λ consists of three components: (1) the Euclidean distances from the target border to all other voxels inside and outside the target, (2) a second array of Euclidean distances from the target border to all voxels outside, and (3) angular distances from a ray passing through the centers‐of‐mass of a selected OAR and terminating on the target centers‐of‐mass. As in Technique 1, the slice‐wise centers‐of‐mass are considered, and the extent in the z‐dimension is restricted to slices containing both the target and the OAR with a three‐slice margin above and below. If there are multiple targets, as in the HN cases, only the high‐risk target (e.g., the target to which the highest dose is prescribed) is used.

Normalizations between [0, 1] using only values within the patient's anatomy are applied to all three components, which are then fit to a sigmoid‐like distribution (Equation 5) with varying parameters. The first Euclidean distance array (component 1) uses fwhm=0.01 and β=15 to provide a narrow band in which the high‐dose conformality to the target will be uniformly reduced by the kernel. The second Euclidean distance array (component 2) uses fwhm=0.05 and β=10, and the angular distance array (component 3) uses fwhm=0.2 and β=10. These values were determined experimentally to provide realistic results.

To provide a larger region of dose decrease relating to overly sparing an OAR, components 2 and 3 are voxel‐wise weighted summed (Equation 6). If more than one OAR is contributing, the individual features are then combined as a voxel‐wise minimum (Equation 7). The overall geometric feature (Equation 10) is a weighted voxel‐wise sum of the distance‐based component providing the narrow band around the target (denoted $\rho_{PTV}$) and the output of the previous step consisting of both distance and angular components (denoted $\lambda_{PTV\_OAR\;}$), where the weighting value of 0.4 applied to $\rho_{PTV}$ was experimentally determined to produce realistic results. An example for a GYN case is shown in Figure 1(b).

Equation 10: Generation of the final geometric feature for reducing target conformality. λ: the final geometric feature, used to reduce target conformality. $\rho_{PTV}$: the distance‐based geometric features for the target, fit to a sigmoid‐like distribution. $\lambda_{PTV\_OAR}$: the geometric feature for the target and OAR, fit to a sigmoid‐like distribution.

(10) $$\lambda =0.4\cdot \rho_{PTV}+\lambda_{PTV\_OAR}$$

The kernel itself is a (1×3×3) array in the z‐, y‐, and x‐dimensions, respectively. The receptive field k(λ) is described by Equation 11.

Equation 11: k(λ): receptive field for the kernel used to decrease PTV conformality. λ: geometric feature.

(11) $$k\;\left(\lambda \right)=\left[\begin{array}{ccc} 1-\lambda & 1-\lambda & 1-\lambda \\ 1-\lambda & 1 & 1-\lambda \\ 1-\lambda & 1-\lambda & 1-\lambda \end{array}\right]$$

For both HN and GYN cases, the minimum scaling values a=0.99,0.98,0.97,0.96,and0.95 were used to generate dose distributions that correspond to approximately 1% to 5% decreases to dose values near the target.

In this study, reductions in PTV conformality due to overly sparing OARs were investigated in groups of one or more OARs. Groupings were made based on geometric or dosimetric relevance and with clinician guidance. The same five HN and three GYN OAR groups were selected as in Technique 1.

From the initial 39 HN cases, 835 suboptimal dose distributions with reduced PTV conformality were generated across the five scaling parameters and five OAR groups. From the initial 20 GYN cases, 290 suboptimal dose distributions with reduced PTV conformality were generated across the five scaling parameters and three OAR groups.

### 2.5 Technique 3: Generate hotspots in target

Unlike Techniques 1 and 2, Technique 3 does not employ geometrically aware convolutions to generate hotspots. Instead, hotspots are simulated as regions of random size and location in the high‐dose target.

To generate hotspots, random walks are initiated within the target mask. The walks are biased 90% in the x‐y plane as this was found to give a realistic shape. A scaling factor is applied so that all values within the random walk have values greater than 1, with all values outside being set equal to 1. To spread out the resultant hotspots, dilation and smoothing are applied (kernel sizes (1×3×3) and (1×5×5), respectively, in the z‐, y‐, and x‐dimensions). Voxel‐wise multiplication of this array with the dose distribution results in hotspots within the target.

In this study, one to four hotspots were randomly generated for each dose distribution, with the random walk size equal to 75 divided by the number of hotspots. Scaling values from 1.07 to 1.13 were randomly selected for each hotspot, corresponding to approximately 7% to 13% dose increases. In total, 39 dose distributions with hotspots in the PTV were generated for the HN disease site and 20 for the GYN disease site.

### 2.6 Comparison of high‐quality and edited dose distributions

To assess changes in the dose delivered to OARs in Technique 1, the relative changes to the mean and maximum dose delivered to OARs is calculated as:

$$\Delta D_{mean}=\frac{{D^{\prime }}_{mean}-D_{0,mean}}{D_{0,mean}}$$

and

$$\Delta D_{\max}=\frac{{D^{\prime }}_{\max}-D_{0,\max}}{D_{0,\max}}$$

where $D^{\prime }$ refers to the dose from the newly generated suboptimal dose distributions and $D_{0}$ refers to the dose from the original predicted high‐quality dose distributions. The choice of mean or maximum dose metrics follows clinical practice at our institution and similar values in the literature.^12^, ^13^

To measure the change in PTV conformality, relative reduction in PTV coverage at the 99% and 95% prescription dose levels were calculated as:

$$\Delta V_{99\%}=\frac{{V^{\prime }}_{99\%}-V_{0,99\%}}{V_{0,99\%}}$$

and

$$\Delta V_{95\%}=\frac{{V^{\prime }}_{95\%}-V_{0,95\%}}{V_{0,95\%}}$$

where $V^{\prime }$ refers to the target coverage in the newly generated, suboptimal dose distributions and $V_{0}$ refers to the target coverage in the original predicted high‐quality dose distributions. The selection of these volume metrics follows clinical practice at our institution and similar values in the literature.^14^

To measure the impact of hotspots, the absolute increase in volume receiving 107% of the prescription dose is calculated as:

$$\Delta V_{107\%}=V_{107\%}^{\prime }-V_{0,107\%}$$

Here, absolute values in cm^3^ are reported, as the high‐quality predictions exhibited very low or zero $V_{107\%}$ values so that relative comparison was not feasible. The metric $V_{107\%}$ was selected based on clinician input and reflects the lower limit of the dose scaling factor applied in our technique.

Testing was conducted to assess whether changes were statistically significant. Normality of the distributions could not be assumed as determined by a Shapiro‐Wilks test,^15^ so the non‐parametric Wilcoxon rank‐sum test^16^ was used. One‐sided testing was conducted with alternate hypotheses as follows for each technique:

1. The $\Delta D_{mean}$ and $\Delta D_{max}$ distributions are greater than zero, indicating that the dose delivered to the OARs was increased.
2. The $\Delta V_{99\%}$ and $\Delta V_{95\%}$ distributions are less than zero, indicating that the volume of the PTV receiving the appropriate dose was decreased.
3. The $\Delta V_{107\%}$ distribution is greater than zero, indicating that the volumes within the PTV receiving excessive dose were increased.

In all cases, changes were considered statistically significant if p<0.05. Testing was conducted over the entire set of generated suboptimal dose distributions.

In addition, experienced clinicians reviewed a subset of the suboptimal dose distributions to provide a qualitative assessment of realism. Two dosimetrists, two physicians, and two physicists reviewed a total of 135 suboptimal dose distributions: 75 with reduced OAR sparing (38 HN, 37 GYN), 40 for reduced PTV conformality (20 HN, 20 GYN), and 20 with hotspots (10 HN, 10 GYN). All examples were randomly selected with equal representation across scaling factors, when applicable.

To assess realism, the randomly selected suboptimal dose distributions were first imported into the RayStation Treatment Planning System (TPS; RaySearch Laboratories, Stockholm, Sweden). For standardization in the review process, dose color wash was turned off and isodose lines were selected based on templates used in clinical review and the prescription dose. Dose distributions were presented in the “Plan evaluation” display window; and although clinicians had the ability to navigate to other windows, none did so. Clinicians were prompted to assess the realism of each dose distribution on a five‐point Likert scale^17^ (1, appears very unrealistic; 2, appears non‐realistic; 3, unsure or ambiguous; 4, appears more realistic than not; 5, appears realistic). Realism was defined as the similarity of the dose distribution to what might be produced by treatment planners of varying skill levels, based upon visual and dosimetric features.

## 3 RESULTS

**FIGURE 2:**
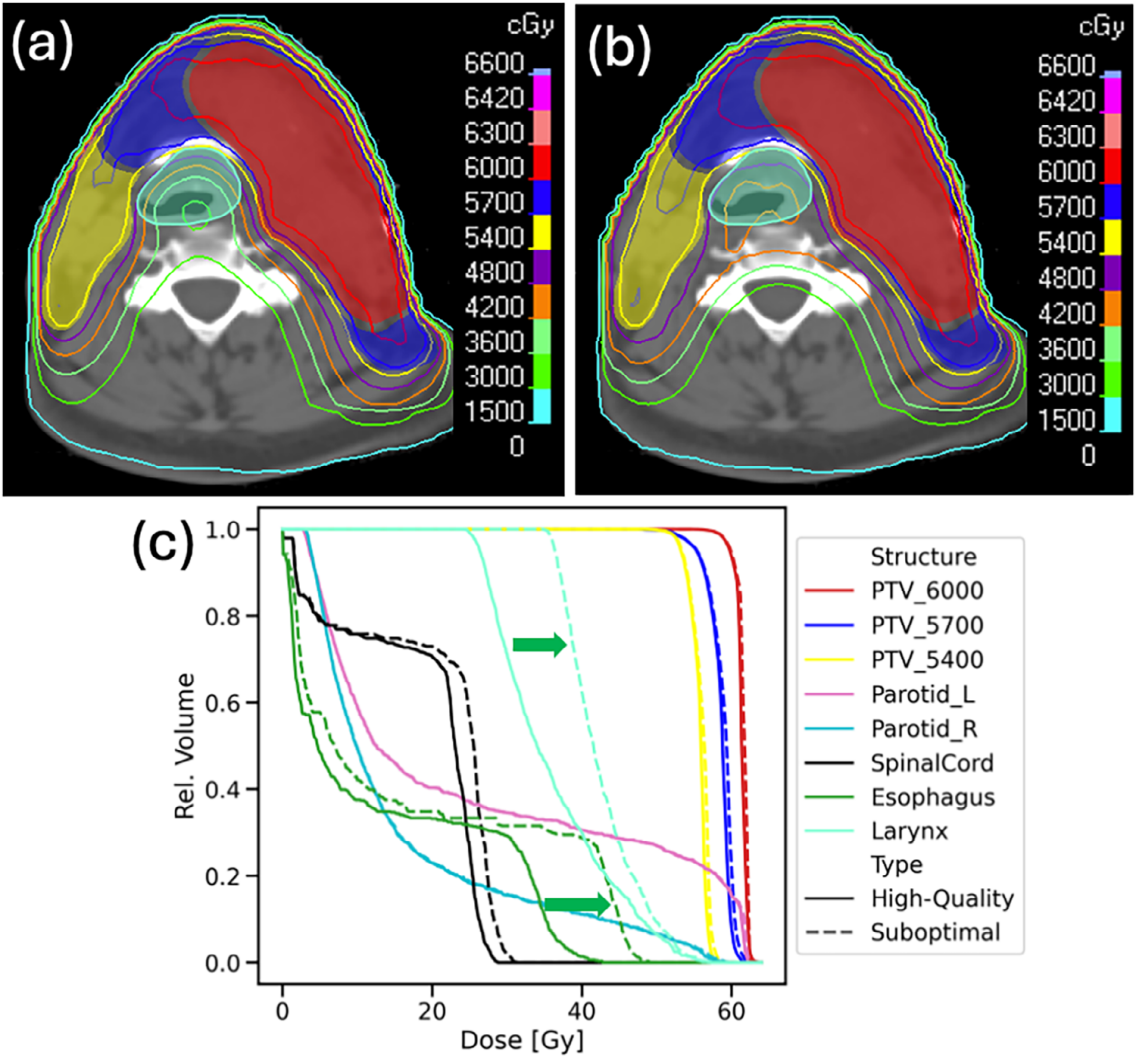
Example of reduction of esophagus and larynx sparing. (a) High‐quality dose distribution prediction. (b) Suboptimal dose distribution. Red, blue, and yellow filled contours: high‐, mid‐, and low‐risk targets. Light green contour: larynx. (c) The dose‐volume histogram (DVH) curves show the substantial increase in the dose to the esophagus and larynx (green arrows). Solid lines: DVH for the high‐quality dose prediction. Dashed lines: DVH for the suboptimal dose distribution.

**FIGURE 3:**
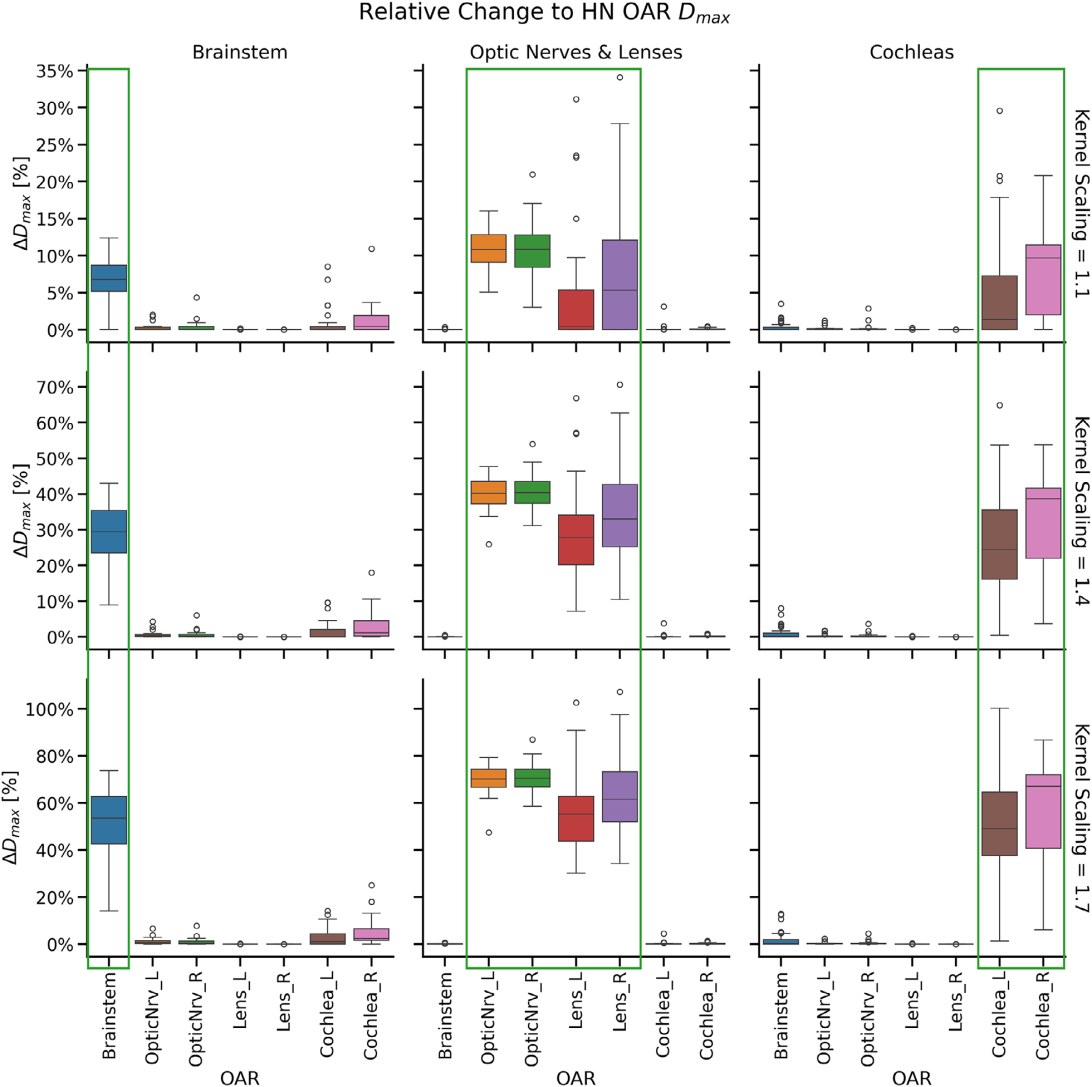
Demonstration of relative change to D_max_ for selected organ at risk (OAR) groups in the head and neck (HN) treatment plans, with selected kernel scaling values.

**FIGURE 4:**
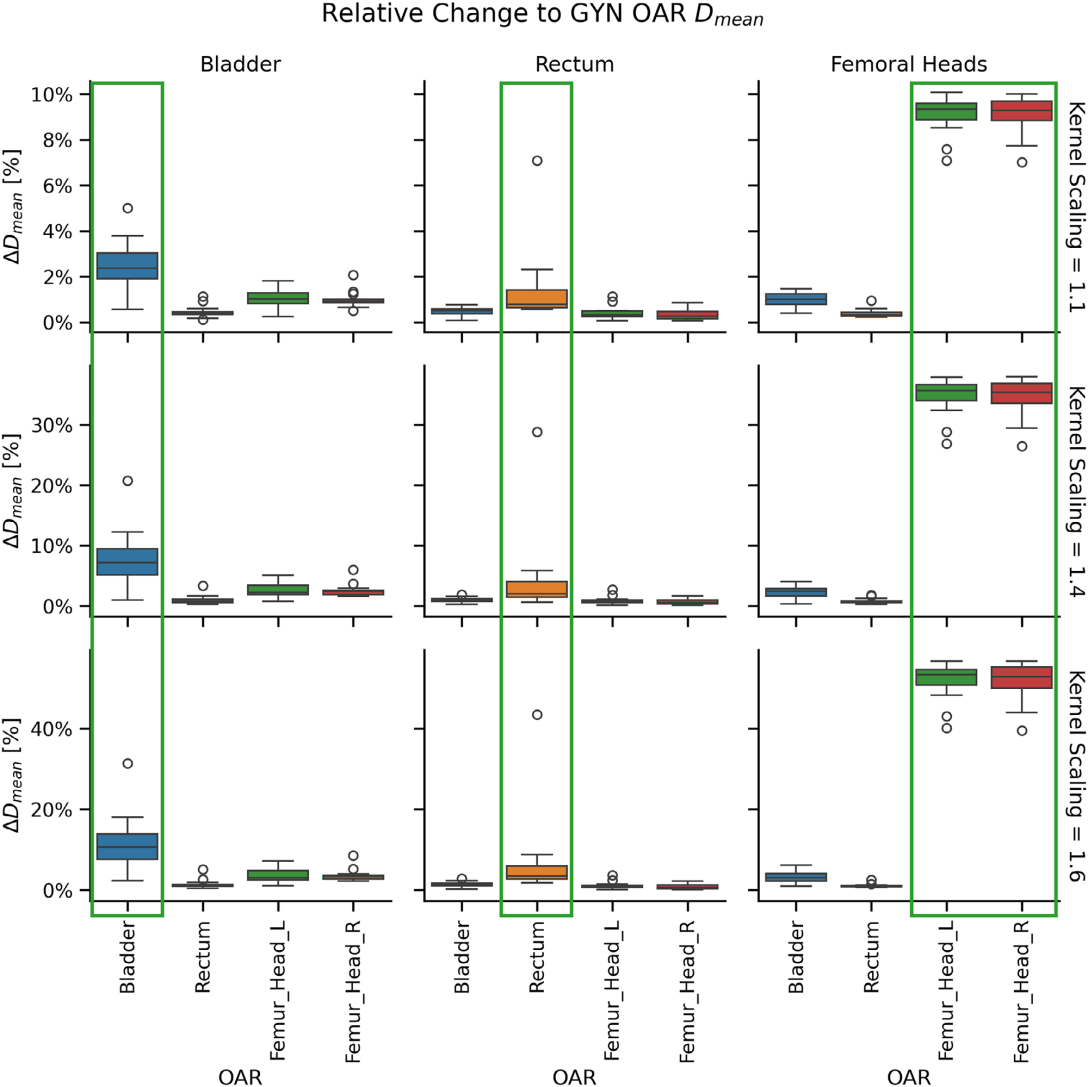
Demonstration of relative change to D_mean_ for selected organ at risk (OAR) groups in the gynecological treatment plans, with selected kernel scaling values.

OAR sparing was successfully reduced with Technique 1. An example of the initial high‐quality dose distributions and the final suboptimal dose distributions in the TPS is shown in Figure 2. Localization of dose increase was preserved, with the OARs in each selected group receiving the greatest relative increase to dose, as seen in Figure 3 and Figure 4. As expected, the relative dose changes increased as the scaling parameter increased. All increases to OAR dose metrics were statistically significant (*p* < 0.05).

**FIGURE 5:**
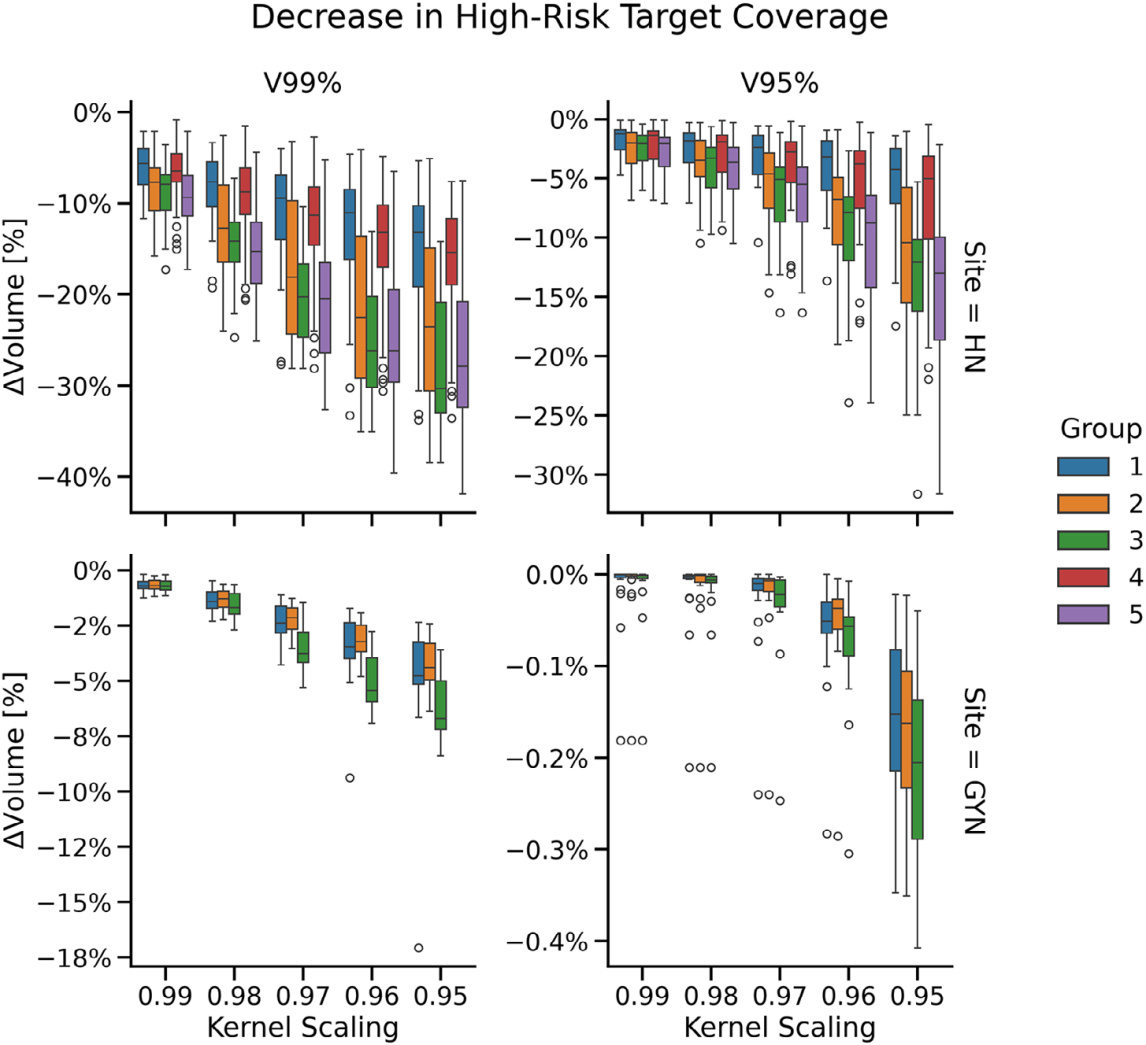
Decrease to V99% and V95% target coverage metrics relative to the high‐quality dose distribution prediction for the high‐risk planning target volume (PTV; head and neck cases [HN]) and the PTV receiving 4500 cGy (gynecological cases [GYN]). Group labels indicate the OARs for which simulated over‐sparing results in reduced target coverage. For HN, these are (1) esophagus and larynx, (2) brainstem, (3) optic nerves and lenses, (4) parotids, and (5) cochleae. For GYN, these are (1) bladder, (2) rectum, and (3) femoral heads.

The conformality and coverage of the high dose to the high‐risk target (HN cases) or single target (GYN) were successfully reduced with Technique 2 (Figure 5). Here, the decreasing target coverage corresponded with decreasing scaling parameter. All changes to volumetric metrics were statistically significant (*p* < 0.05).

**FIGURE 6:**
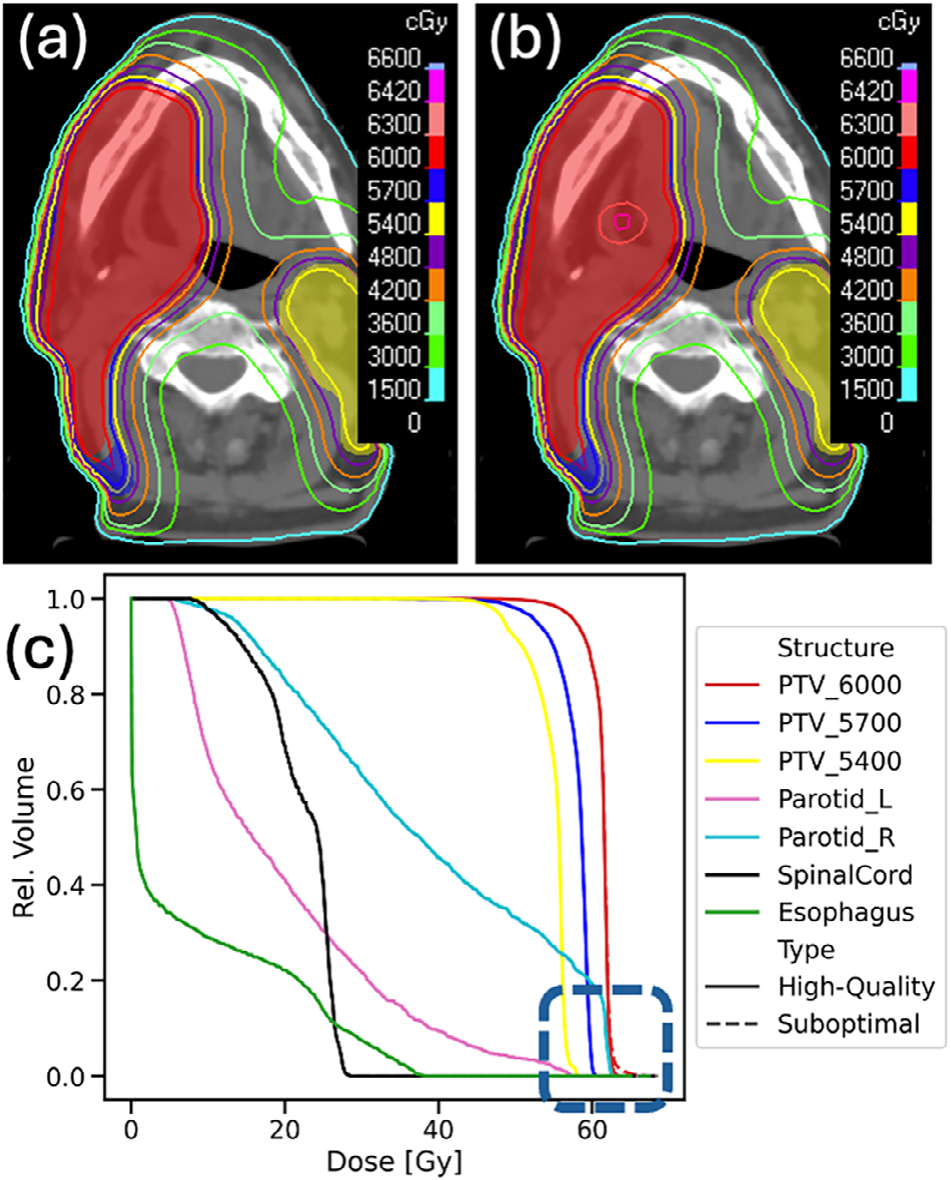
Example of introducing a hotspot to the high‐risk target. (a) High‐quality dose distribution prediction. (b) Suboptimal dose distribution. Red and yellow filled contours: high‐ and low‐risk targets. (c) The dose increase to a small volume of the target can be seen in the dose‐volume histogram (DVH) curve (blue dashed box). Solid lines: DVH for the high‐quality dose prediction. Dashed lines: DVH for the suboptimal dose distribution.

Hotspots in the high‐risk target (HN cases) or single target (GYN cases) were successfully introduced with Technique 3. All changes were statistically significant (*p* < 0.05). An example of the initial high‐quality dose distributions and the final suboptimal dose distributions in the TPS is shown in Figure 6.

Clinical review showed a split between disciplines. The number of scores greater than or equal to 4 (“appears more realistic than not”) or 3 (“unsure or ambiguous”) is shown in Table 1. Except for physicist review of the HN plans, a majority of plans appeared either realistic or ambiguous.

**TABLE 1:** Clinician scoring of dose distribution realism. The ratios of examples meeting or exceeding Likert score 4 (“appears more realistic than not”) and 3 (“unsure or ambiguous”) to total examples shown to each clinician reviewer, as well as the percent meeting or exceeding each score, is shown below.

	HN		GYN	
	≥4 (%)	≥3 (%)	≥4 (%)	≥3 (%)
Physician	17/19 (89%)	17/19 (89%)	15/19 (79%)	19/19 (100%)
Physicist	6/21 (29%)	10/21 (48%)	11/21 (52%)	15/21 (71%)
Dosimetrist	8/28 (29%)	15/28 (54%)	20/27 (74%)	23/27 (85%)

## 4 DISCUSSION

In this work, three techniques were developed to simulate suboptimal radiotherapy plans by starting with a high‐quality predicted dose distribution and then adding errors to (1) reduce OAR sparing, (2) reduce target conformality, or (3) introduce hotspots into the target. The techniques operate directly on dose distributions and are fully controllable both in the region into which errors are added and the magnitude of change to the dose distributions. To the best of our knowledge, this is the first work to deliberately introduce errors into dose distributions on this scale and with the goal of creating examples for clinical education.

A major benefit of this approach is the relative computational simplicity. The techniques are easily understandable and can be modified by adjusting a few parameters, rather than more advanced techniques such as deep learning‐based approaches that are “black boxes” and would require retraining to adjust. Furthermore, they can easily run on ordinary desktop hardware, such as that found in a typical clinic.

Additionally, these techniques allow the generation of a large training dataset from a few examples. In this work, over 2800 new examples were created from only 59 dose distributions. While deep learning predictions of high‐quality dose distributions were used as the starting examples in this work, the techniques are applicable to dose distributions from other sources, such as VMAT treatment plans from online archives or institutional databases. This can help address the challenge to effective education posed by the limited presentation of different disease types at many clinics (unpublished National Cancer Database analysis, 2021), as a wide range of training examples can be generated from only a few cases and those cases can originate from a variety of sources.

The computational simplicity and flexibility also underscore an important methodological distinction. While the term “convolution” is used throughout this work, the geometrically aware convolutions are not components of the pre‐trained deep learning model used to generate the initial high‐quality dose distributions, which remained unchanged. Instead, the geometric convolutions were implemented independently and applied to introduce controlled degradations in plan quality. These operations are not part of any neural network and could equivalently be applied to dose distributions derived from clinical plans or other sources, without any involvement of deep learning.

The level of control for dose change is illustrated in Figure 3 and Figure 4 for the reduced OAR sparing scenario. As the kernel scaling parameter increases, the dose to selected OARs increases accordingly, simulating greater deviation from ideal sparing. The spatial extent of dose increase is also well controlled: OARs targeted for reduced sparing (highlighted in green boxes) receive substantially more dose than other structures. This demonstrates precise control over both the location and magnitude of dose changes. Figure 5 shows similar control in reducing target coverage, where reducing kernel scaling values leads to progressively lower target coverage as assessed by V99% and V95%. These demonstrate that the proposed techniques can simulate a wide range of clinically relevant deviations, from subtle to severe. This flexibility supports progressive training, allowing educators to tailor examples to different levels of learner experience. By enabling controlled exposure to realistic suboptimal plans, the approach directly addresses gaps in current training environments.

When reducing OAR sparing with Technique 1, the magnitude of dose increase to the OAR will be reduced if the OAR is partly or fully encompassed within the PTV in the treatment plan. This occurs due to the limitations imposed on dose increase within the PTV to prevent unrealistic hotspot generation. In the current study, this was noted particularly for the rectum and bladder (Figure 4); however, the increase in dose to these structures remained statistically significant. Similarly, reducing OAR sparing is challenging for OARs near the target or targets. For example, in Figure 1(a), the values of the geometric feature for the anterior aspect of the right parotid were higher than for other regions within the right parotid or equivalent regions in the left parotid, which was more distal to the target. This resulted in relatively less increase in dose to the anterior aspect of the right parotid. This compromise was necessary to avoid introducing visibly unrealistic hotspots outside the target.

During study design, care was taken to choose appropriate parameters (e.g., the range of kernel scaling values) to produced dose distributions that appeared visually and dosimetrically similar to machine‐deliverable plans. As this work is intended to produce examples of suboptimal dose distributions for clinical training, which should never be used for plan delivery, developing fully deliverable plans was not a requirement of this study. Furthermore, the clinician review evaluated the realism of the generated dose distributions with respect to plans originating in a TPS. This approach supports the development of critical evaluation skills by presenting a spectrum of realistic, though not necessarily deliverable, scenarios that mirror the types of errors encountered in clinical practice.

There are some limitations associated with the current work. The introduction of errors outside a TPS meant that the cascading effects that changing the dose delivered to a particular structure has on the other structures was not fully modeled. For this study, the ability to directly introduce errors was prioritized for interpretability and application in education. Future research could explore the use of a TPS‐based approach, or a deep learning model trained directly on suboptimal plans, to model more complex effects.

Another limitation of this study is the absence of direct comparison between the simulated suboptimal dose distributions and suboptimal clinical plans. Such comparisons are challenging due to the lack of standardized definitions for suboptimality and the variability in clinical practice across institutions. Instead, the expert clinician review served to assess the similarity between the suboptimal dose distributions and actual plans. Nonetheless, future work could incorporate curated datasets of clinically identified suboptimal plans to further evaluate and refine the realism and educational value of the simulated examples.

In addition to these technical considerations, the clinical review process provided further insight into the realism and limitations of the generated dose distributions. During review, low scores were frequently assigned to dose distributions based on features introduced by the initial dose prediction models. In particular, all clinical reviewers believed that the model for high‐quality dose distributions did not model the low dose isodose lines very well. This highlights a limitation of the current study: the realism of the suboptimal dose distributions depends not only on the techniques described in this work but also on the initial dose distribution to which errors are introduced. In fact, the two failing examples in the radiation oncologist HN review were due to errors in the initial dose distributions, specifically where the starting dose distributions were not felt to be physically deliverable. However, the techniques will work on any VMAT dose distribution, so this can easily be addressed through choice of alternative methods to generate the initial dose distributions. It is additionally worth highlighting that radiation oncologists typically felt the dose distributions were realistic. This provides increased confidence that these techniques can be integrated into the education of radiation oncology residents.

Finally, the realism assessment relied on a small group of reviewers, and inter‐rater reliability was not formally evaluated. While the goal was to capture diverse perspectives across disciplines, future studies could include a larger and more diverse reviewer pool agreement to strengthen the validity of qualitative assessments.

## 5 CONCLUSION

Residents have identified gaps in their educational processes that diminish their confidence in assessing and improving radiotherapy plan quality, potentially leading to diminished patient outcomes. To address the pitfalls of example availability and clinical time pressures in the typical educational environment in which plan review is taught, the current work introduces multiple techniques to simulate suboptimal plans by introducing errors into dose distributions. The location and magnitude of errors are fully controllable, and the resultant dose distributions appear realistic to experienced clinicians. Potential applications include integration of these suboptimal dose distributions into the plan review curriculum of radiation oncology residents and other clinical trainees.

## AUTHOR CONTRIBUTIONS

**Skylar S. Gay**: Conceptualization, Methodology, Software, Validation, Formal Analysis, Investigation, Resources, Writing — Original Draft, Writing — Review and Editing, Visualization. **Mary P. Gronberg**: Methodology, Software, Writing — Review and Editing, Visualization. **Raymond Mumme**: Software, Data Curation, Writing — Review and Editing. **Beth M. Beadle**: Investigation, Writing — Review and Editing. **Anuja Jhingran**: Investigation, Writing — Review and Editing. **Tze Yee Lim**: Investigation, Writing — Review and Editing. **Zhiqian H. Yu**: Investigation, Writing — Review and Editing. **Christine Chung**: Investigation, Writing — Review and Editing. **Meena Khan**: Investigation, Writing — Review and Editing. **Chelsea Pinnix**: Conceptualization, Methodology, Writing — Review and Editing, Funding Acquisition. **Sanjay Shete**: Conceptualization, Methodology, Writing — Review and Editing. **Brent Parker**: Conceptualization, Methodology, Writing — Review and Editing. **Tucker J. Netherton**: Conceptualization, Methodology, Writing — Review and Editing. **Carlos E. Cardenas**: Conceptualization, Methodology, Writing — Review and Editing. **Laurence E. Court**: Conceptualization, Investigation, Methodology, Writing — Review and Editing, Supervision, Project administration, Funding acquisition.

## CONFLICT OF INTEREST STATEMENT

This work was supported by a grant from The University of Texas MD Anderson Cancer Center, Division of Radiation Oncology. Skylar Gay received support from the Cancer Answers Scholarship, the Larry Deaven PhD Fellowship in Biomedical Sciences, and the Dr. John J. Kopchick Fellowship. There are no other conflicts of interest to disclose.
